# Evaluation of factors predicting postoperative recurrence and metastasis of parathyroid cancer: a single-center study

**DOI:** 10.1186/s12957-023-02912-2

**Published:** 2023-02-03

**Authors:** Keiko Ohkuwa, Ryohei Katoh, Kiminori Sugino, Mitsuji Nagahama, Wataru Kitagawa, Kenichi Matsuzu, Nobuhiro Fukunari, Koichi Ito

**Affiliations:** 1 Department of Surgery and Thyroid Center, Showa University Northern Yokohama Hospital, Kanagawa, Japan; 2 Department of Surgery, Ito Hospital, Tokyo, 150-8308 Japan; 3 Department of Pathology, Ito Hospital, Tokyo, Japan

**Keywords:** Parathyroid carcinoma, Ki-67, Metastasis, Recurrence, Prognosis

## Abstract

**Purpose:**

The purpose of this study was to examine the postoperative clinical course of parathyroid carcinoma to determine factors that predict postoperative recurrence and distant metastasis.

**Methods:**

In this retrospective study, we included 38 patients with parathyroid carcinoma who received surgical intervention at Itoh Hospital between 1979 and 2020. Clinicopathologic characteristics (age, sex, intact PTH, serum Ca level, operation type, parathyroid weight, parathyroid size, histopathologic findings: vascular invasion, capsular invasion, necrosis, histological type, and Ki-67 staining) were used. The median follow-up observation period was 63.7 months.

**Results:**

Postoperatively, 5 patients (13.2%) developed distant metastasis or had localized recurrence, and 3 patients died (7.9%). The results of the univariate analysis revealed three factors affecting distant metastasis and recurrence, which were Ki-67 (*p* = 0.0041), the presence or absence of necrosis (*p* = 0.0163), and tumor weight (*p* = 00,189). Using the cutoff values obtained by ROC analysis, which were 4.1 for Ki-67 (sensitivity of 80% and specificity of 96.9%) and 4890 mg for tumor weight (sensitivity of 100% and specificity of 60.9%), we calculated the cumulative incidence of recurrence and distant metastasis by the three factors retained. We found that the presence of the three factors was associated with a high possibility of distant metastasis or recurrence during the 5-year follow-up period.

**Conclusions:**

Three factors, Ki-67, necrosis, and tumor weight in parathyroid carcinoma, may predict outcomes of postoperative recurrence and distant metastasis.

## Introduction

Parathyroid carcinoma (PC) is a rare endocrine tumor accounting for 0.5–6% or less in cases of primary hyperparathyroidism [1–3]. Currently, the World Health Organization (WHO) histopathologic findings are the standard methods for diagnosing the malignancy of parathyroid neoplasms. According to the above, the histopathologic criteria for PC include the presence of vascular invasion with capsular invasion and progression and/or metastasis to adjacent tissues [4]. However, the small number of cases of PC makes it difficult to predict prognosis in the postoperative period of PC.

The 5- and 10-year overall survival (OS) rates for PC patients are estimated to be 78–85% and 49–70%, respectively [5–7]. Parathyroid cancer is an endocrine tumor with poor prognosis presenting severe clinical findings such as recurrence and metastasis. Histopathologic criteria of parathyroid cancer proposed by the World Health Organization (WHO) alone are insufficient to predict the degree of malignancy, such as of postoperative recurrence and distal metastasis. Recently, a nationwide survey examined prognostic factors in parathyroid carcinoma; Kong et al. reported that three factors were prognostic as follows: age > 50 years, parathyroidectomy only, and previous reoperation [8]. Other reports indicate that tumor size, distant metastasis, and other factors define prognosis [9]. However, because of the small number of cases of parathyroid cancer at a single institution and the paucity of reports, even in multi-institutional studies, there is a need to examine factors that predict prognosis in the postoperative period of parathyroid cancer.

Characteristics of PC include severe hypercalcemia, palpable mass in the cervical region, intraoperative findings of localized invasion, and suspicion of distant metastasis by preoperative examination [3, 10, 11]. Aspiration biopsy cytology of the parathyroid gland for diagnosis carries the risk of causing cell dissemination, and therefore, as a rule, it is not recommended [12, 13]. In general, parathyroid carcinoma and parathyroid adenoma are difficult to distinguish before surgery because the clinical presentation, biochemical findings, and imaging findings are almost identical to each other; hence, most patients are presumed to have a benign parathyroid adenoma, and a surgery is performed on them. It is also difficult to distinguish the parathyroid carcinoma from the parathyroid adenoma at the time of surgical resection. Consequently, it is not uncommon for cancer to be diagnosed by histopathologic examination after surgery [1, 14]. Most patients who develop localized recurrence and distant metastasis die due to uncontrollable severe hypercalcemia [5, 15]. Therefore, curative treatment involves performing total resection by surgery and reliably removing the lesion, making it undoubtedly the only desired treatment [3, 15–17]. When postoperative recurrence occurs, surgical resection has been reported to have prolonged survival [18]. Reoperation at recurrence is therefore an effective approach.

Following surgery, once PC has been diagnosed by histopathologic diagnosis, the goal in the management of PC is to detect recurrence of the disease early and to reduce death by aggressive treatment. In actual clinical practice, some cases are encountered that have good progress with no recurrence and no distant metastasis. Therefore, predictors of recurrence and/or distant metastasis are needed to distinguish between cases that follow a slow course and those that should be followed closely. To distinguish cases of such gradual progression and cases with severe progression that should be observed, accurate risk stratification of PC is needed. We hypothesized that by searching for prognostic predictors of recurrence and/or distant metastasis based on clinicopathologic examination results, we could identify cases that require aggressive postoperative management. Therefore, we aimed to examine factors that serve as indicators to determine prognosis following surgery for PC.

## Material and methods

### Study population

Among 1521 patients with primary hyperparathyroidism who underwent surgery at Ito Hospital between 1979 and 2020, we retrospectively examined the data of 38 patients (2.5%) with PC confirmed by postoperative histopathology. Surgery was performed either by resection of the parathyroid lesion only or by en bloc surgery, which was defined as resection of the parathyroid lesion with or without thyroidectomy and ipsilateral central lymph node dissection. In all patients, the PC diagnosis was confirmed by pathologists in accordance with the histopathological diagnostic criteria of the WHO [19]. Histopathologic characteristics of PC include vascular invasion, capsular invasion, invasion into adjacent organs, and metastasis. For all patients, clinical data (age, sex, serum calcium, parathyroid hormone (intact PTH), and tumor size) was collected from medical records. For the size of the parathyroid gland, we used the maximum value obtained by preoperative cervical ultrasound examination. Data such as disease-free survival (DFS) and overall survival (OS) were collected from outpatient clinic records or during follow-up. Surgery was performed with curative intention. The patients did not undergo any additional procedures or experimental investigations.

### Immunohistochemical staining

Immunohistochemical staining was performed on sections prepared from formalin-fixed and paraffin-embedded tissue with the highest density of cancer cells. Preserved sections were deparaffinized with xylene and rehydrated with alcohol. Endogenous peroxidase was blocked by incubation with 3% hydrogen peroxide. To unmask the antigens, microwave heating was performed for 10 min with 10 mM sodium citrate (pH6). Thereafter, nonspecific staining was blocked with phosphate-buffered saline containing 3% bovine serum albumin. After blocking, the sections were incubated with Ki-67 (1:200 dilution, clone MIB-1 (Dako, Glostrup, Denmark)). After washing, the prepared slides were incubated with biotin-labeled secondary antibodies.

The MIB-1 index was defined as the percentage of cells with positive nuclei in a tumor section using e-Count2, cell counting software (e-Path, Kanagawa, Japan). Images of tumor sections mounted on glass slides were converted to JPEG (Joint Photographic Experts Group) format, and cells with positive immunostaining for Ki-67 were count based on pixel color intensity. Images were automatically segmented into Ki-67-positive and Ki-67-negative areas according to the pixel color intensity cutoff point. The median number of tumor cells counted was about 1000 (range, 823–1102) in each sample.

### Statistical analysis

Quantitative variables are expressed as median, and qualitative variables are expressed as numerical values and percentages. The difference between the median of each group was compared using the Kruskal–Wallis test. The relationship between various factors and the status of recurrence was analyzed by logistic regression analysis, and Cox proportional hazards model was used to conjecture prognostic factors that affect recurrence taking time into account. The Kaplan–Meier method was used to calculate the cumulative incidence, and a log-rank test was used to test for a significant difference. Furthermore, the cutoff values of the factors extracted from the Cox proportional hazards analysis were calculated from the sensitivity and specificity using receiver operating characteristic (hereinafter referred to as ROC) curves. *P* < 0.05 was considered statistically significant. As the statistical analysis software, JMP (version 12, SAS Institute Inc., Cary, NC, USA) was used.

### Postoperative follow-up observation

Follow-up examination included blood tests (parathyroid hormone levels and serum calcium levels) and diagnostic imaging such as ultrasound, which were performed once or twice per year. A CT scan was also performed to search for distant metastases.

### Ethics statement

This study was approved by the Ethics Committee of Ito Hospital. The study was conducted in accordance with the guidelines described in the Declaration of Helsinki, and consent was obtained after providing verbal explanation of the study to the patients.

## Results

### Clinical characteristics of parathyroid carcinoma

**Fig. 1:**
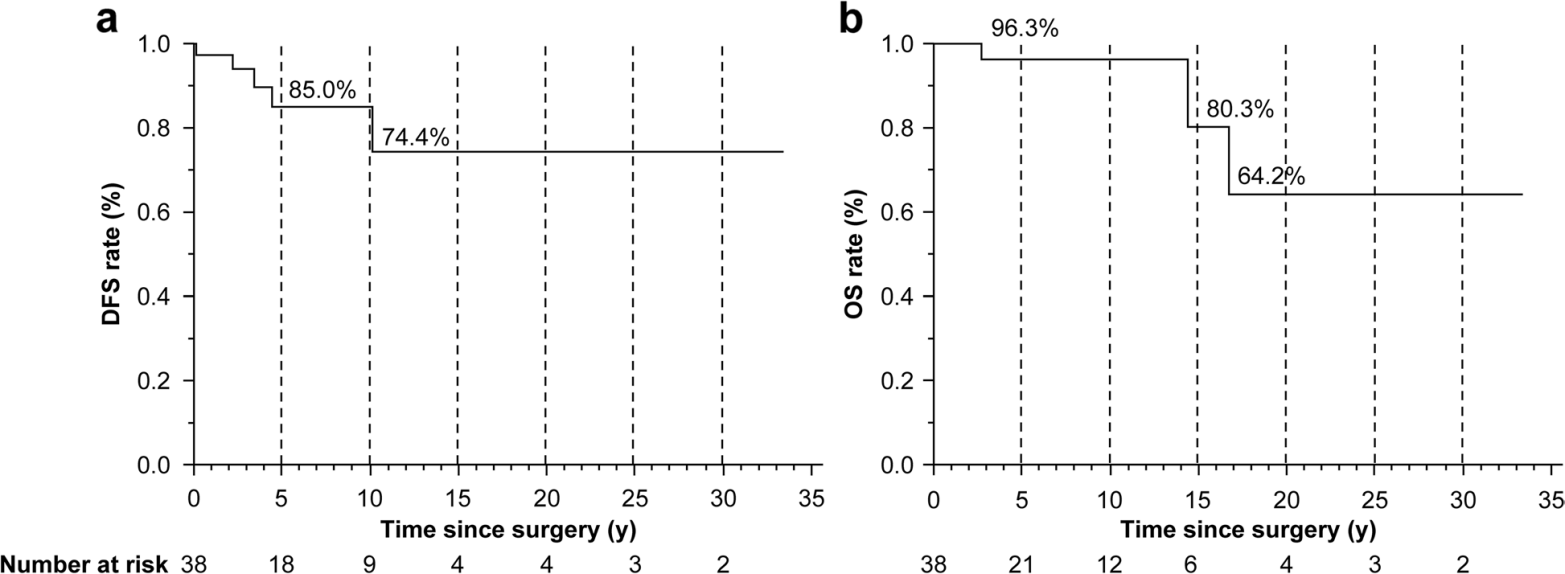
Kaplan–Meier curves of DFS and OS for parathyroid carcinoma patients. DFS, disease-free survival; OS, overall survival

There were 1521 patients with primary hyperparathyroidism who underwent surgery at Ito Hospital between 1979 and 2020. Among these, 38 patients with PC diagnosed by postoperative histological diagnosis were included in the analysis. Table 1 presents the background of patients in the present study. The median age at surgery was 56.5 years. The median tumor length on preoperative cervical ultrasound was 29 mm. The initial procedure was parathyroid adenomectomy alone in 17 patients (44.7%) and resection of the adenoma and thyroidectomy in 21 patients (55.3%), among who, regional lymph node dissection was performed in 13 patients (34.2%), and in approximately half of the patients, the parathyroid gland and surrounding organs (thyroid gland and lymph nodes) were resected at the time of the initial surgery. Pathological characteristics included capsular invasion in 92.1%, with vascular invasion in 55.3%, and the majority of component cells were chief cells. Additional postoperative treatment included external beam radiation therapy in 2 patients. Postoperative recurrence was observed in 5 patients (13.2%), and tumor-specific death occurred in 3 patients. The median follow-up observation period was 63.7 months. The overall survival (OS) and disease-free survival (DFS) for all patients are shown in Fig. 1. In the present study, the OS at 5 years and 10 years was 96.3%.

**Table 1:** Clinicopathological characteristics of patients with parathyroid carcinoma

**Characteristic**	**Number/median**	**%/[IQR]**
No. of patients	38	
Age, year	56.5	[46.0, 67.5]
Sex		
Male	11	28.9%
Female	27	71.1%
Preoperative calcium (mg/dl)	11.5	[10.9, 12.8]
Preoperative intact PTH (pg/ml)	252	[163.5, 370.6]
Tumor size, mm	29	[17, 35.5]
Surgical procedure		
Parathyroidectomy only	17	44.7%
Parathyroidectomy + thyroid lobectomy	8	21.0%
Parathyroidectomy + thyroid lobectomy + lymph node dissection	13	34.3%
Postoperative radiotherapy		
Yes	2	5.2%
No	36	94.8%
Histological component cells		
Chief cells	31	81.6%
Oxyphil cells	7	18.4%
Capsular invasion		
Yes	35	92.1%
No	3	7.9%
Vascular invasion		
Yes	21	55.3%
No	17	44.7%
Histological necrosis		
Yes	5	13.2%
No	33	86.8%
Lymph node metastasis		
Yes	1	2.6%
No	12	31.6%
Not dissecting	25	65.8%
Weight of tumor (mg)	3930	[1180, 8070]
Ki-67 index (%)	0.875	[0.35, 1.75]
Postoperative course		
Local recurrence + distant metastasis	1	2.6%
Distant metastasis	3	7.9%
Ongoing hypercalcemia	1	2.6%
Tumor-specific death		
Yes	3	7.9%
No	35	92.1%
Follow-up time (months)	63.7	[31.6, 127.9]

Data are expressed as number (%) or median (interquartile range/IQR). *PTH*, parathyroid hormone; normal range; intact PTH, 15–65 pg/ml; calcium, 8.5–10.0 mg/dl

### Analysis of prognostic factors

The results of our comparison of clinicopathologic characteristics according to the status of postoperative recurrence and distant metastasis are presented in Table 2. In the group with postoperative recurrence and distant metastasis, tumor weight was predominantly greater (*p* = 0.0343), and necrosis was observed in the pathological findings (*p* = 0.0108). The Ki-67 index was predominantly higher in the group with postoperative recurrence and distant metastasis (*p* = 0.00197). Histopathologic result revealed no significant difference in terms of capsular invasion findings and vascular invasion findings.

**Table 2:** Comparison of clinicopathological factors in the presence or absence for metastasis or recurrence of parathyroid carcinoma

	**Absent *n* = 33**		**Present *n* = 5**		
	**Number/median**	**%/[IQR]**	**Number/median**	**%/[IQR]**	***p*-value**
Age, y	53	[47, 65]	57.0	[45.5, 68]	NS
Sex, male/female	9/24	27.3/72.7	2/3	40.0/60.0	NS
Preoperative serum calcium level, mg/dl	11.4	[10.7, 12.7]	11.6	[11.1, 15.9]	NS
Preoperative intact PTH, pg/ml	253.0	[171.4, 330.4]	195.0	[102.5, 1243]	NS
Tumor size, mm	28.0	[16.5, 35.7]	35.0	[25, 40]	NS
Surgical procedure					
Parathyroidectomy/en bloc	15/18	45.5/54.5	2/3	40.0/60.0	NS
Lymph node dissection, yes/no	11/22	66.7/33.3	2/3	40.0/60.0	NS
Postoperative radiotherapy, yes/no	1/32	3.0/97.0	1/4	20.0/80.0	NS
Tumor weight, mg	1800	[1180, 8010]	16,735	[5560, 28950]	0.0343
Histological component, chief cell/oxyphil cell	27/6	81.8/18.2	4/1	80.0/20.0	NS
Capsular invasion, +	30	90.9	5	100.0	NS
Vascular invasion, +	17	51.5	4	80.0	NS
Necrosis, +	2	6.1	3	60.0	0.0108
Ki-67 index, %	0.8	[0.35, 1.44]	4.2	[2.22, 8.65]	0.0197

*PTH* parathyroid hormone, *IQR* interquartile range, *NS* not significant

The univariate analysis results of clinicopathologic factors that affect postoperative metastasis and distant metastasis are presented in Table 3. Three factors, including Ki-67, necrosis, and tumor weight, were considered significant prognostic factors of recurrence and distant metastasis following surgery for PC. To evaluate the prognostic value of Ki-67 and tumor weight, we conducted calculations based on the presence or absence of postoperative recurrence and distant metastasis using a ROC curve. As per the results, the cutoff value for Ki-67 was 4.1% with an area under the ROC curve (AUC) of 0.8273. The cutoff value for tumor weight was 4890 mg with an AUC of 0.8370. The sensitivity and specificity were 80.0% and 96.9%, respectively, when the cutoff value of Ki-67 was 4.1%. Furthermore, at a tumor weight cutoff of 4890 mg, the sensitivity and specificity were 100% and 60.9%, respectively.

**Table 3:** Univariate Cox proportional hazards regression models to evaluate clinicopathological factors as predictors of distant metastasis and recurrence

**Variable**	**Category**	**HR**	**95% *CI***	***p*-value**
Age, year^a^		1.00	0.93–1.07	0.9904
Sex	Female	Reference		
	Male	1.50	0.19–9.15	0.6617
Preoperative serum calcium level, mg/dl^a^		1.67	0.97–3.43	0.0661
Preoperative intact PTH, pg/ml^a^		1.00	0.99–1.00	0.1429
Surgery	Parathyroidectomy alone	Reference		
	Combined thyroidectomy and/or Ln dissection	0.96	0.09–0.12	0.9649
Tumor weight, mg^a^		1.00	1.00–1.001	**0.0189**
Tumor diameter, mm^a^		1.00	0.94–1.05	0.9885
Vascular invasion	Absent	Reference		
	Present	3.47	0.51–68.06	0.2159
Necrosis	Absent	Reference		
	Present	9.45	1.56–71.64	**0.0163**
Histological component cells	Chief cells	Reference		
	Oxyphil cells	1.17	0.16–23.24	0.8911
Ki-67 index, %^a^		1.46	1.14–1.98	**0.0041**

*CI* confidence interval, *HR* hazard ratio

^a^Continuous variables

**Fig. 2:**
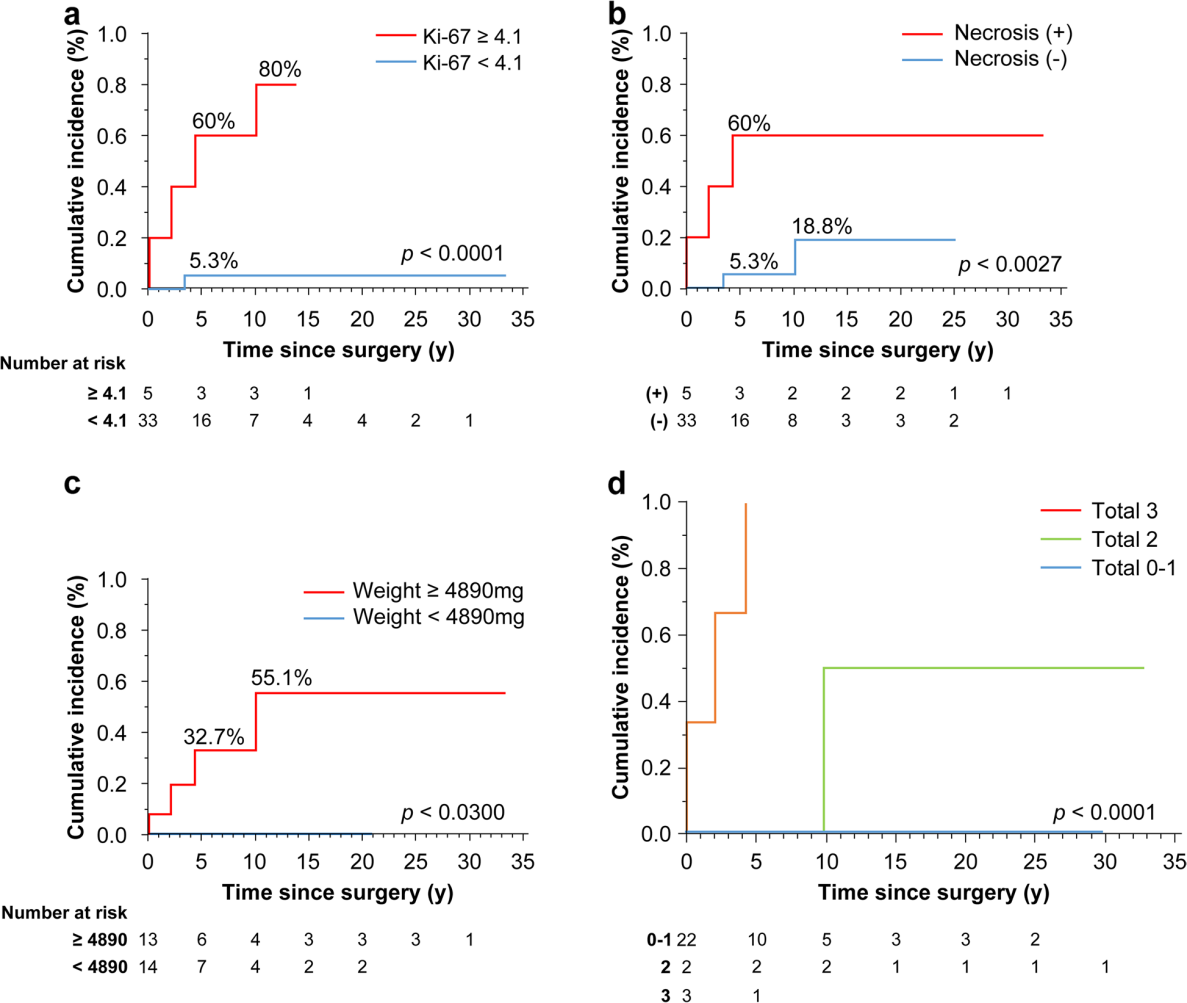
Cumulative incidence of recurrence and distant metastasis. **a** Ki-67. **b** Necrosis findings. **c** Tumor weight

Figure 2 presents the cumulative incidences of postoperative recurrence and distant metastasis. Patients with Ki-67 ≥ 4.1% were more likely to develop recurrence or distant metastasis earlier than those with Ki-67 < 4.1% (Fig. 2a). Patients with pathologic necrosis were more likely to develop recurrence or distant metastasis earlier than those without necrosis (Fig. 2b). Furthermore, patients with tumor weight ≥ 4890 mg had no recurrence or distant metastasis compared to those with tumor weight < 4890 mg (Fig. 2c).

Furthermore, we stratified the three factors (Ki-67, necrosis. and tumor weight) according to the corresponding number of cells and examined the cumulative incidences of postoperative recurrence and distant metastasis (Fig. 2d). In the group with the three factors, postoperative recurrence and distant metastasis developed within 5 years. By combining Ki-67, necrosis, and tumor weight, we obtained results with high prognosis predictive power (*p* < 0.001).

## Discussion

Determining the prognosis of PC accurately and effectively is clinically significant for subsequent follow-up. However, owing to the rarity of PC, there is little clinical evidence regarding prognosis, and because there is no approved staging system, clinicians have difficulty determining prognosis accurately. However, we experienced cases that presented gradual clinical findings only and that progressed postoperatively without recurrence and metastasis. According to research in recent years using a population-based database, several reports have investigated prognostic factors of PC patients. Sadler et al. analyzed the National Cancer Data Base (NCDB) and found that lymph node metastasis positivity and age are associated with reduced survival rate [16]. Like Sadler et al., Asare et al. analyzed the NCDB and reported that patient age, tumor size, and sex affect the survival of PC patients [20]. Although the two reports used the same database, we believe that a difference arose in the analysis results because the subject samples included different age groups.

Definitive diagnosis of PC can only be made by histological examination following surgical resection. Schantz and Castleman first reported the pathological criteria defining PC in 1973 [21], the characteristics of which included capsular invasion, vascular invasion, fibrous bands, and mitotic activity. Thereafter, according to the WHO classification that was revised in 2017, the diagnosis of malignant tumors should be limited to the following: (1) tumors showing invasive proliferation into the thyroid gland and soft tissue with confirmed capsular and/or extracapsular vascular invasion and metastasis, (2) vascular invasion occurring when the tumor invades the capillary vessels or surrounding soft tissue vessels, and (3) tumor cell properties are often similar, with cell populations formed by broad bands of fibrous connective tissue extending from the capsule surrounding the tumor. Zonal histology is present in 90% of PCs and mitotic images in 80%; however, both characteristics are not considered to be specific to malignant tumors [19]. In immunohistological staining, Ki-67 of 5% or above suggests PC [22–24]. In terms of prognosis, Kameyama et al. reported elevated Ki-67 levels in PC patients with a poor prognosis [25], and Iihara et al. suggested that Ki-67 of 5% or above indicates a poor prognosis, and in particular, that recurrence develops early after surgery when Ki-67 is 10% or above [26]. Moreover, Lenshow et al. demonstrated that a Ki-67 index of 10% increases the postoperative recurrence rate% [27]. In our study also, we confirmed that the Ki-67 index is a prognostic factor for recurrence and metastasis. Patients with a Ki-67 index of 4.1% or higher had a higher incidence of recurrence and distant metastasis than those with a Ki-67 index of less than 4.1% (*p* < 0.001). With regard to the Ki-67 index, though our results were lower than the cutoff value previously reported, we believe that this may be because we used the occurrence of recurrence or distant metastasis as a criterion in this study in order to examine the indicators for early detection of recurrence and individualized treatment, while the previous report used the survival rate.

Bondeson et al. suggested that findings of coagulation necrosis in pathological findings are related to recurrence of PC [28]. Williams et al. proposed a dataset for PC as a record for future guidelines [29]. They noted that necrosis might be more generalized in tumors of high-grade malignancy and incorporated necrosis as a required element for such guidelines. However, as a point of caution, necrosis should be differentiated from nonspecific infarct-like necrosis, which might be caused by spontaneous infarction, prior episodes of rupture, and preoperative cytodiagnosis. In the present study, patients with necrosis, compared to patients without necrosis, exhibited a higher incidence of recurrence and distant metastasis, thereby demonstrating the usefulness of the findings of necrosis (*p* < 0.0027).

With regard to the effect of the tumor size on the prognosis of PC, the literature offers different opinions. Asare et al. reported that tumor size is a predictor of distant metastasis, and that the development of distant metastasis affects survival [30]. Lee et al. and Talat et al. reported that tumor size is not an important prognostic marker [3, 6]. On the other hand, Hsu et al. concluded that a tumor size of 30 mm or greater is associated with lymph node metastasis [31]. Furthermore, Asare et al. found a correlation between patients with tumor size of 40 mm or greater and an increased risk of death [20], while Qian et al. reported that among 604 patients with PC in the Surveillance, Epidemiology and End Results (SEER) database, tumor size of 35 mm or greater is associated with worsening of cancer-specific survival (CSS) in patients with PC [9]. In our analysis, there was no significant difference in parathyroid adenoma size, but in parathyroid weight, the results were consistent with the possibility of recurrence or distant metastasis after surgery for PC. Furthermore, in the present study, as a result of having examined the tumor weight measured postoperatively using the cutoff value of 4890 mg from the ROC analysis as a predictor of prognosis (sensitivity 100% and specificity 60.9%), patients with tumor weight ≥ 4890 mg, compared to those with < 4890 mg, had a higher incidence of recurrence and distant metastasis (*p* = 0.0300). Few reports in the past have examined tumor weight. The reason for this is that when PC is suspected preoperatively, the tumor is resected along with the surrounding organs, especially the thyroid gland, to account for the possibility of disease spread to the surrounding parathyroid tissue. Therefore, as the results of the present study were determined once the parathyroid gland alone was weighed and only serve as a reference, not all cases of PC can necessarily be determined preoperatively, and thus, in cases where tumor weight has been measured, we believe that it is useful as supporting diagnosis of prognosis.

The purpose of the present study was to investigator indicators that can serve to predict prognosis in patients with PC diagnosed postoperatively by histopathologic diagnosis. We examined the incidence of recurrence and distant metastasis according to the number of patients with the risk factors identified in the present study, including Ki-67, pathological findings of necrosis, and tumor weight. As a result, the higher the number of patients with these risk factors, the higher the incidence of recurrence and distant metastasis, and in actual clinical practice, it was possible to identify patients who needed careful follow-up observation. We believe that it will serve as an indicator for early detection and treatment of postoperative recurrence and metastasis of parathyroid cancer and will help improve the prognosis by early treatment.

The present study has several limitations. First, the study is a single-center analysis, which limits the number of cases. Second, uncontrolled or unrecognized bias due to the relatively small sample size is inevitable. Accordingly, to further evaluate the clinical applicability of the present results, a further prospective study is needed by multicenter collaborative research with a larger sample size. However, the fact that a prospective study of extremely rare tumors is not possible, we believe, it is also a limitation of this study. 

## Conclusions

The present study demonstrated that there are three factors that predict recurrence and prognosis in patients with PC diagnosed postoperatively by histopathological diagnosis. These three factors were Ki-67 index, weight of the parathyroid gland, and findings of necrosis. Patients with Ki-67 index ≥ 4.1, parathyroid gland weight ≥ 4890 mg, and necrosis findings might develop recurrence and distant metastasis early and require strict follow-up. We believe that the factors above are useful as indicators to predict prognosis following surgery and can help select appropriate treatment.

## Data Availability

The datasets generated during and/or analyzed during the current study are available from the corresponding author on reasonable request.
